# Low Leakage Current and High Breakdown Field AlGaN/GaN MIS-HEMTs Using PECVD-SiN*x* as a Gate Dielectric

**DOI:** 10.3390/mi13091396

**Published:** 2022-08-26

**Authors:** Xiaohui Gao, Hui Guo, Rui Wang, Danfeng Pan, Peng Chen, Dunjun Chen, Hai Lu, Rong Zhang, Youdou Zheng

**Affiliations:** 1 Jiangsu Provincial Key Laboratory of Advanced Photonic and Electronic Materials, School of Electronic Science and Engineering, Nanjing University, Nanjing 210093, China; 2 Microfabrication and Integration Technology Center, Nanjing University, Nanjing 210023, China

**Keywords:** PECVD-SiN*x*, AlGaN/GaN, MIS-HEMTs, in situ plasma treatment, breakdown field

## Abstract

In this paper, SiN*x* film deposited by plasma-enhanced chemical vapor deposition was employed as a gate dielectric of AlGaN/GaN high electron mobility transistors (HEMTs). We found that the NH_3_ flow during the deposition of SiN*x* can significantly affect the performances of metal–insulator–semiconductor (MIS) HEMTs. Compared to that without using NH_3_ flow, the device with the optimized NH_3_ flow exhibited three orders of magnitude lower gate leakage current, two orders of magnitude higher ON/OF drain current ratio, and an increased breakdown field by 69%. In addition, an in situ N_2_ plasma surface treatment prepared prior to SiN*x* deposition can further improve DC performances of MIS-HEMTs to a very low gate leakage current of 10^−9^ mA/mm and a high ON/OFF drain current ratio up to 10^9^ by reducing the interface state density. These results demonstrate the great potential for using PECVD-SiN*x* as a gate dielectric in GaN-based MIS-HEMTs.

## 1. Introduction

Gallium nitride-based HEMTs have been considered excellent candidates for next-generation high-efficiency power switching devices due to their remarkable material and transport properties. However, conventional Schottky-gate HEMTs have a large gate leakage current and a small gate swing, which are limited by the Schottky-gate forward turn-on voltage (e.g., <3 V) [1,2]. To mitigate these issues, MIS-HEMTs have been proposed. It can suppress gate leakage and improve gate reliability by inserting an insulator between the gate metal and the barrier layer. Various dielectric materials are suitable as gate insulators, such as SiO_2_ [3], SiN*x* [4,5,6,7,8,9,10], Al_2_O_3_ [11,12], and SiON [13]. Among them, nitride-based gate dielectrics (e.g., SiN*x*) can avoid the additional introduction of interfacial Ga-O bonds, which tend to introduce interface traps and cause threshold voltage instability [4,14,15,16,17]. Currently, SiN*x* has been widely used as a gate dielectric/interfacial gate dielectric. There are many methods to deposit SiN*x*, such as plasma-enhanced chemical vapor deposition (PECVD) [1,18], low-pressure chemical vapor deposition (LPCVD) [4,5,6,8], and metal–organic chemical vapor deposition (MOCVD) [7,9].

Deposition methods, deposition conditions, and surface treatment before dielectric deposition can significantly affect the properties of SiN*x* films. Recently, LPCVD has been reported as a possible alternative technique for depositing high-quality SiN*x* [4,5,6,8]. However, dielectric deposition at a very high temperature is not compatible with typical microelectronic interconnect processing and may create surface defects due to the decomposition of GaN above 650 °C [19]. In situ SiN*x* grown by MOCVD may have Si contamination in the MOCVD chamber [5]. Compared with these gate dielectric deposition methods, using plasma-enhanced chemical vapor deposition (PECVD) SiN*x* shows great advantages in low temperature, easy operation, and high deposition rate [20,21,22]. PECVD-grown SiN*x* has been proven to be an effective material to suppress current collapse and reduce the surface states in AlGaN/GaN HEMTs.

In this work, we investigated the influence of SiN*x* deposition condition and surface treatment on the electrical properties of GaN-based MIS-HEMTs employing PECVD-SiN*x* as the gate dielectric. To evaluate the dielectric quality and interface properties, current–voltage (I–V) characterization, X-ray photoelectron spectroscopy (XPS), and capacitance–voltage (C–V) measurements were performed. The results showed that the optimized device exhibits a lower leakage current, a higher ON/OFF drain current, and a higher breakdown field than the device without any optimization.

## 2. Device Fabrication

**Figure 1:**
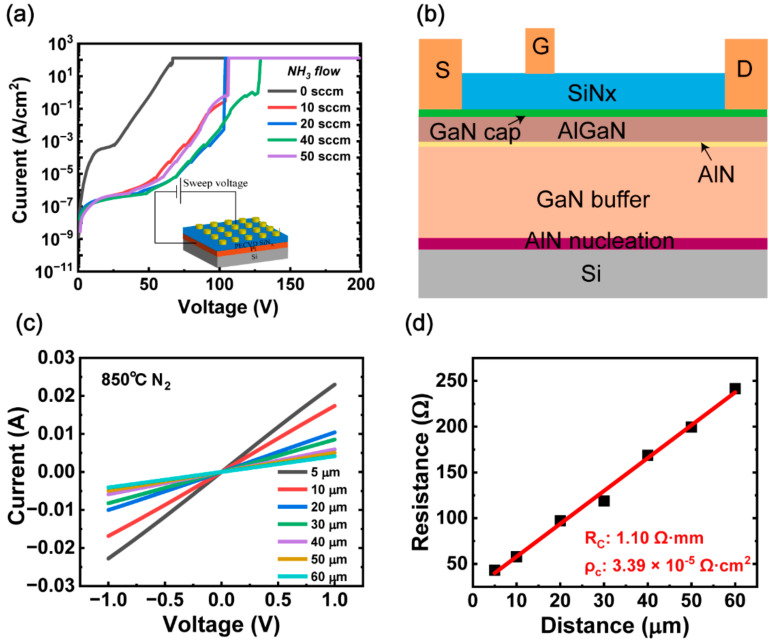
(**a**) Forward leakage current characteristics for circular MIM patterns with a 100 μm diameter. (**b**) Schematic cross–sectional view of fabricated PECVD-SiN*x* MIS-HEMTs. (**c**) I–V characteristics of a TLM structure of Ti/Al/Ni/Au contacts after annealing at 850 °C in N_2_ ambient. (**d**) Extracted resistance versus TLM distance.

To improve the breakdown field of the SiN*x* dielectric, we first optimized the deposition conditions of SiN*x* on Pt substrates using plasma-enhanced chemical vapor deposition by changing the flow of NH_3_. The reactant precursors were SiH_4_ (5%)/N_2_, NH_3_ and N_2_. The as-deposited 100 nm SiN*x* films were fabricated into metal–semiconductor–metal (MIM) capacitors for the measurements of breakdown field strength. The flow of NH_3_ was varied with a fixed SiH_4_: N_2_ ratio, a chamber pressure (=600 mtorr), and an RF power of 10 W. The results showed that NH_3_ flow could significantly affect the breakdown field of SiN*x* films, as presented in Figure 1a, and finally a maximum breakdown field of 11.45 MV/cm, which exceeds the reported breakdown field strength of PECVD-SiN*x* as a gate dielectric, was obtained at the condition of NH_3_ flow of 40 sccm.

The AlGaN/GaN heterostructure was grown by MOCVD on a silicon substrate. The epitaxial structure consisted of a 1-nm GaN cap layer, a 15-nm AlGaN barrier layer, a 1-nm AlN layer, a 4-µm GaN buffer layer, and an AlN nucleation layer, as shown schematically in Figure 1b. The use of AlN nucleation layer can release the tensile stress and reduce the dislocation defects on the epitaxial surface of GaN, thus improving the crystalline quality of GaN and reducing the cracking [23,24]. The device-fabrication started with electron beam evaporation of Ti/Al/Ni/Au and alloyed at 850 °C for 30 s in a N_2_ atmosphere to form an ohmic contact. The value of specific contact resistivity (ρ_c_) of the ohmic contact was 3.39 × 10^−5^ Ω·cm^2^, as seen in Figure 1c,d [25,26]. Subsequently, a mesa isolation process was implemented by an inductively coupled plasma (ICP) system using a BCl_3_/Cl_2_ gas mixture. After that, 30-nm SiN*x* under the condition without NH_3_ gas was deposited for sample A and 30-nm SiN*x* under the condition with 40 sccm NH_3_ was deposited for sample B. Considering the effect of surface treatment, an in situ N_2_ plasma treatment before SiN*x* deposition, with NH_3_ gas flow of 40 sccm, was employed for sample C. The Ni/Au (50/100 nm) gate metal was then deposited by E-beam evaporation. Finally, the contact window was opened by a reactive ion etching (RIE) dry-etching system. The gate-to-source space is 4 µm, the gate-to-drain space is 12 µm, and the gate width and length are 100 µm and 4 µm, respectively. The fabricated devices were marked device A, B, and C, corresponding sample A, B, and C, respectively.

## 3. Results and Discussion

### 3.1. Material Optimization Characteristics

**Figure 2:**
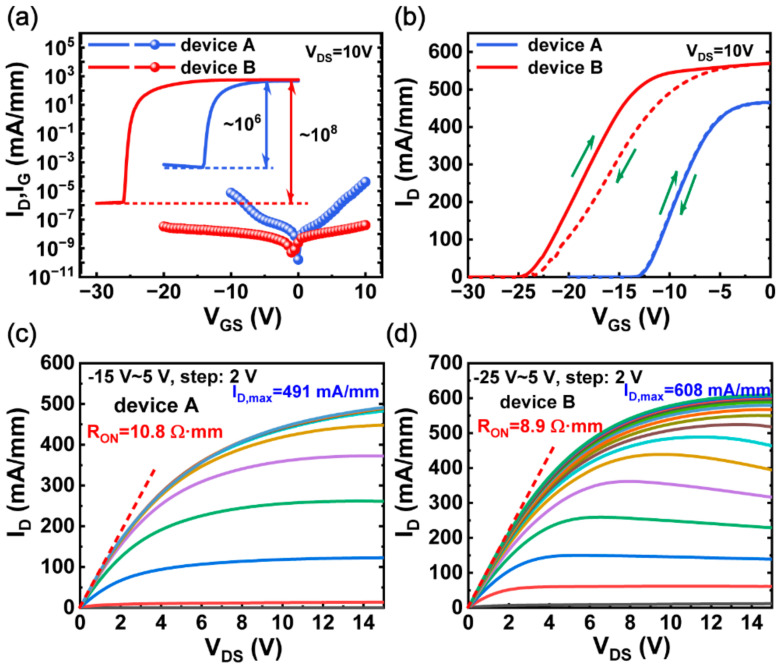
(**a**) Transfer and gate leakage characteristics of device A and B on a log scale. (**b**) Transfer characteristics of device A and B on a linear scale. Output characteristics of (**c**) device A and (**d**) device B. Device dimensions: L_GS_/L_GD_/L_G_/W_G_ = 4/12/4/100 µm.

Figure 2 shows typical DC transfer, gate leakage, and output characteristics of PECVD-SiN*x*/AlGaN/GaN MIS-HEMTs. The transfer characteristics of MIS-HEMTs were measured at a drain bias voltage of 10 V. Compared with device A, device B exhibits a much higher ON/OFF drain current ratio of ~10^8^. In addition, device B shows a very low gate leakage current of ~10^−8^ mA/mm at both V_G_ = −20 V and V_G_ = 10 V, three orders of magnitude lower than that of device A, suggesting that SiN*x* deposition with NH_3_ flow can effectively suppress leakage paths in the gate dielectric. In Figure 2b, device B shows a relatively larger threshold voltage hysteresis, compared with device A. This indicates that SiN*x* without NH_3_ can alleviate the surface trapping effect. Meanwhile, a large negative threshold voltage shift in device B may come from the high density of positive charges existing at the interface between SiN*x* and AlGaN [27]. In addition, these positive charges may be introduced by the hydrogen radicals originating from NH_3_. As shown in Figure 2c,d, device B shows a larger saturated output current of 608 mA/mm and a lower on-resistance of 8.9 Ω·mm, compared with device A.

**Figure 3:**
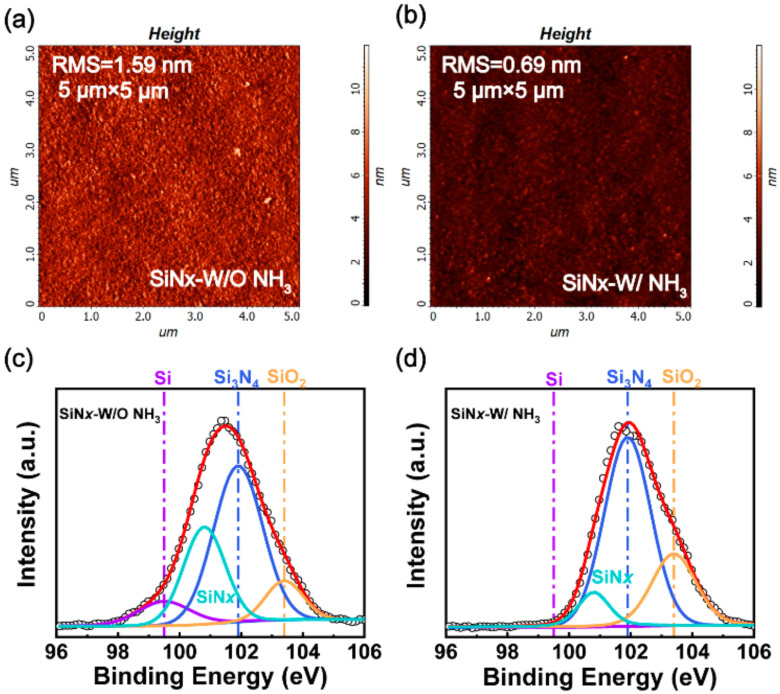
AFM images of the SiN*x* surface in (**a**) device A and (**b**) device B within a 5 µm × 5 µm area. Fitting XPS results of the Si 2p core-level spectrum of (**c**) the SiN*x* film without NH_3_ gas and (**d**) the SiN*x* with an NH3 gas flow of 40 sccm.

To further understand the reasons for the improved electrical performances obtained by optimizing the NH_3_ flow, we analyzed the dielectric surface roughness and the N/Si ratio of SiN*x* with different deposition conditions by employing atomic force microscopy (AFM) and X-ray photoelectron spectroscopy (XPS). As shown in Figure 3a,b, the root mean square (RMS) roughness over a 5 µm × 5 µm area is 1.59 nm for SiN*x* without NH_3_, but 0.69 nm for SiN*x* with 40 sccm NH_3_, indicating that NH_3_ can effectively reduce the surface roughness of the SiN*x* dielectric. Typical Si 2p core-level spectrums were captured as shown in Figure 3c,d. Three components corresponding to Si-Si bonds, Si-N bonds, and Si-O bonds were extracted from the spectrum. The amount of elemental composition incorporation in SiN*x* was estimated in Table 1 by using the standard method, as reported in Ref. [28]. The SiN*x* stoichiometry (*x* = [N]/[Si]) was analyzed by the peak area sensitivity method [29], and the results showed that the corresponding N/Si ratio was changed from 0.75 to 1.05 before and after optimizing NH_3_ flow, respectively. Moreover, with the increase in N/Si ratio, the Si-Si bond contents decreased while the Si-O bond and Si-N bond contents increased. An increase in the N/Si ratio was demonstrated to be able to improve the insulation characteristics of the SiN*x* dielectric film, as reported in Refs. [27,30], which can explain why device B exhibits better DC characteristics than device A.

### 3.2. Interface Optimization Characteristics

**Figure 4:**
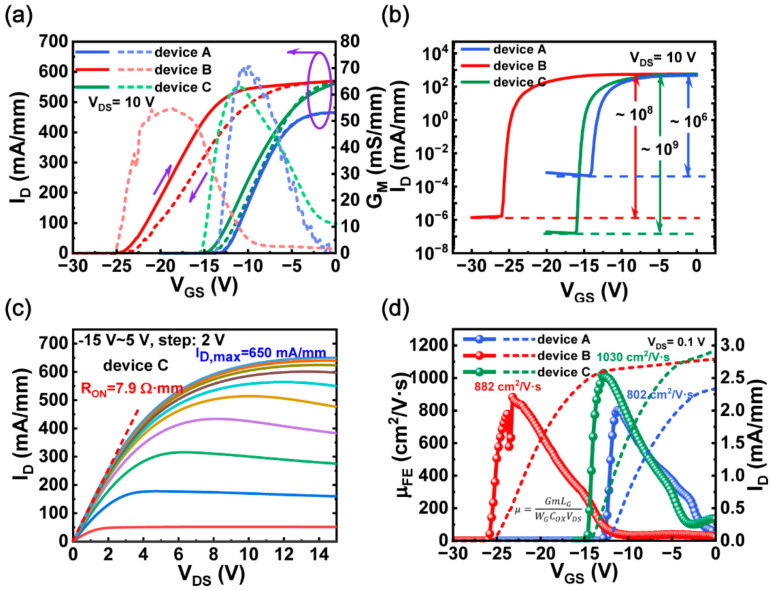
Transfer characteristics of device A, B, and C on the (**a**) linear scale and (**b**) log scale. (**c**) Output characteristics of device C. Device dimensions: L_GS_/L_GD_/L_G_/W_G_ = 4/12/4/100 µm. (**d**) Extracted field–effect mobility using a long–channel device with L_G_/W_G_ = 40/100 µm at V_DS_ = 0.1 V.

However, device B shows a larger voltage hysteresis than device A, which may result from hydrogen radicals originating from NH_3_. It has been confirmed that NH_3_ can diffuse into the GaN structure and react with Ga-N bonds, thus resulting in the generation of nitrogen vacancies and Ga-H bonds near the surface [31,32]. To reduce these interface defects, some surface treatment methods have been well developed [33,34,35,36,37]. In this work, an in situ N_2_ plasma surface treatment was adopted before dielectric deposition for device C which has the same subsequent processes as device B. As shown in Figure 4a,b, device A shows the smallest V_TH_ hysteresis and the lowest ON/OFF drain current ratio. In addition, it can be seen that the saturation current in device A is the smallest, indicating that device A has the lowest 2DEG concentration at the AlGaN/GaN interface. Device C exhibits a large positive shift of V_TH_ compared to device B, which may be related to the reduction of nitrogen-vacancy defects, thus reducing the positive charges [34]. Meanwhile, the transconductance and ON/OFF drain current ratio (~10^9^) of device C are further improved, indicating that in situ N_2_ plasma treatment can suppress the trapping of channel carriers by surface defects. Moreover, device C exhibits the lowest ON-resistance of 7.9 Ω·mm and the highest saturation output current up to 650 mA/mm in comparison with device A and B, which can be attributed to the improvement of field-effect mobility. As presented in Figure 4d, device C shows the highest field-effect mobility because of the improvement in device transconductance, thus reducing the on-resistance in device C. Since the on-resistance is mainly related to field-effect mobility and carrier concentration, device A has the largest on-resistance. Generally, after optimization, the maximum drain current and the field-effect mobility of device C increased by 32% and 28%, respectively, compared with device A.

**Figure 5:**
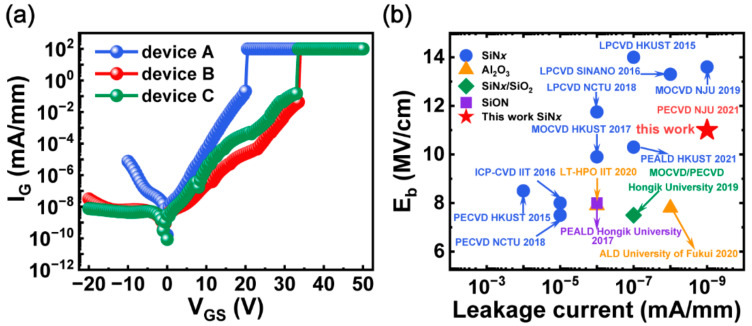
(**a**) I_G_−V_GS_ characteristics of PECVD-SiN*x*/AlGaN/GaN MIS-HEMTs. Device dimensions: L_GS_/L_GD_/L_G_/W_G_ = 4/12/4/100 µm. (**b**) Benchmark of leakage current versus electrical field in recent reports [3,4,5,6,7,8,9,10,11,12,13,38].

Furthermore, the gate leakage characteristics of the MIS-HEMTs were measured at V_DS_ = 0 V as shown in Figure 5. In comparison with device A, device B and C exhibit much lower gate leakage current and higher forward gate breakdown voltage up to 34 V at room temperature due to the optimized deposition condition of the SiN*x* dielectric with NH_3_ flow. The corresponding electric field strength (E_b_) was estimated to be 11 MV/cm, a very competitive result referring to those of reported MIS-HEMTs. We benchmarked the gate leakage current versus electrical field against these reported AlGaN/GaN MIS-HEMTs [3,4,5,6,7,8,9,10,11,12,13,38], as plotted in Figure 5b. In general, the gate leakage and breakdown field of the optimized device in this work are comparable to those of the reported state-of-the-art AlGaN/GaN MIS-HEMTs.

**Figure 6:**
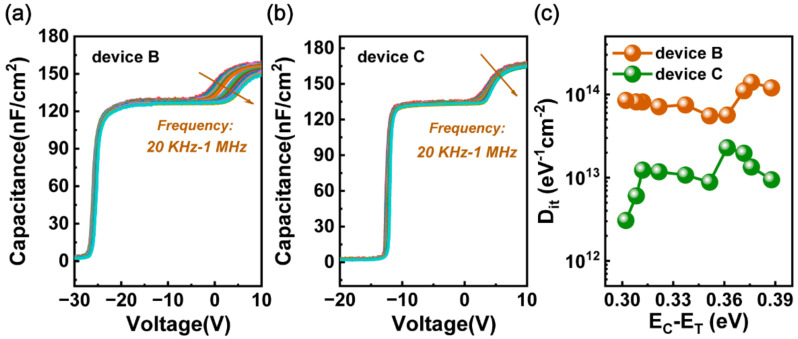
C−V characteristics of (**a**) device B and (**b**) device C with frequencies varying from 20 kHz to 1 MHz. (**c**) Trap density as a function of energy level for device B and C.

Finally, capacitance–voltage (C–V) measurements were performed on the MIS diodes, corresponding to device B and C, with different frequencies varying from 20 kHz to 1 MHz at room temperature. As shown in Figure 6, there are two obvious steps in both C-V curves. The first step corresponds to the process of accumulating two-dimensional electron gas in the AlGaN/GaN heterojunction while the second step reflects the electron transfer from the AlGaN/GaN to the SiN*x*/GaN interfaces [39]. In comparison with device B, device C exhibits a smaller frequency dispersion in the second step, indicating a higher quality interface of SiNx/GaN that has a lower trap density. We also calculated the interface state density using the method in Ref. [40], and the results are shown in Figure 6c. We found that the interface state density was reduced by almost one order of magnitude after the in situ N_2_ plasma surface treatment indicating that nitrogen vacancies and surface oxygen-related bonds can be effectively reduced by an in situ N_2_ plasma treatment, thus obtaining a high-quality SiNx/GaN interface. 

## 4. Conclusions

In summary, we studied the impact of SiN*x* growth condition and surface treatment on the electrical properties of AlGaN/GaN MIS-HEMTs when employing PECVD-SiN*x* as a gate dielectric. The results show that the gate leakage current can be greatly suppressed by increasing the N/Si ratio of SiN*x*, and the interface state density can be obviously reduced through in situ N_2_ plasma surface treatment. The optimized SiN*x*/AlGaN/GaN MIS-HEMTs exhibit low gate leakage current, high I_ON_/I_OFF_ ratio, and high breakdown field. These results prove that PECVD-SiN*x*/AlGaN/GaN MIS-HEMT is comparable to those of the reported state-of-the-art AlGaN/GaN MIS-HEMTs.

## Figures and Tables

**Table 1:** The elemental composition of SiN*x* grown by PECVD.

Samples	Si	N	N/Si
SiN*x*-W/O NH_3_	34.43	25.7	0.75
SiN*x*-W/NH_3_	28.1	29.52	1.05
